# Editorial: Secondary Metabolites and Peptides as Unique Natural Reservoirs of New Therapeutic Leads for Treatment of Cancer and Microbial Infections

**DOI:** 10.3389/fchem.2021.748180

**Published:** 2021-08-12

**Authors:** Maria Luisa Mangoni, Anirban Bhunia, Bruno Botta, Francesca Ghirga

**Affiliations:** ^1^ Department of Biochemical Sciences, Laboratory Affiliated to Istituto Pasteur Italia-Fondazione Cenci Bolognetti, Sapienza University of Rome, Rome, Italy; ^2^ Department of Biophysics, Bose Institute, Kolkata, India; ^3^ Department of Chemistry and Technology of Drugs, “Department of Excellence 2018−2022”, Sapienza University, Rome, Italy

**Keywords:** antimicrobial peptides, secondary metabolite, antimicrobial resistance, cancer, infectious disease

The bleakest outlook for a “post-antibiotic era” is one in which microbial infections can no longer be cured. The traditional antibiotic pipeline has been exhausted, while antimicrobial resistance has become a multifaceted crisis, imposing a serious threat to human health with more than 700,000 deaths each year globally. Furthermore, in line with the latest reports from the World Health Organisation (WHO), this number is expected to grow up to 10-fold by 2050, meaning that infectious diseases will be the principal cause of mortality rather than cancer (Sulis et al., 2021). The growing ineffectiveness of clinically used antibiotics could result in new pandemics that will be difficult to stem without effective replacement drugs. The current SARS-CoV-2 crisis is illuminating with respect to the consequences of being poorly armed to combat infection, and how this impacts all aspects of modern society; medical, social, and economic. From this scenario, it is clear how the discovery of new antimicrobial treatments is highly demanded.

Inspired by nature, antimicrobial peptides (AMPs) and secondary metabolites (SM) are gaining attention for their clinical translation and remain the best storehouse for leads in modern drug discovery. AMPs are evolutionarily conserved elements of the innate immune system of almost all living organisms and have distinct advantages compared to conventional antibiotics. Indeed, they have a large spectrum of antimicrobial activity and cause cell disruption through a non-specific mechanism based on the perturbation of the target microbial cell membrane, thus making microbes less prone to acquire resistance to them (Sarkar et al., 2021). AMPs also display additional functions that indirectly promote the clearance of microorganisms through the modulation of inflammatory host responses and the promotion of wound healing. In addition, some of them have promising anticancer properties. However, some inherent drawbacks of AMPs need to be overcome for their clinical translation. Among them, the short half-life owing to the susceptibility to protease degradation; the inactivity at physiological salt concentrations; the cytotoxicity towards host cells, and the limited targeted delivery. Currently, most of the AMPs under clinical evaluation are positively charged derivatives of naturally occurring AMPs and are limited to topical administration for an effective concentration at the target site (Casciaro et al., 2017).

Plants are a unique source of medicines and traditional remedies since ancient times. Due to their biodiversity, medicinal plants represent the largest library of structurally diverse SM that has ever existed and cannot be matched by any synthetic screening libraries. Several clinically-used therapeutic agents with antimicrobial and/or antitumor activity derive from SM by means of chemical transformations or total syntheses, thus strengthening the pivotal role of nature as a source of hit and lead compounds in drug discovery ( Appendino et al., 2010; Atanasov et al., 2021; Ghirga et al., 2021).

In this Research Topic, we present a collection of original research and review articles that show the ability of phytochemicals and natural peptides to display *in vitro*/*in vivo* activities against tumour cells and/or microbial pathogens and how the employment of computational techniques and/or efficient synthetic tools and nanotechnology approaches offer promising solutions for the improvement of traditional medicine and the production of proper drug-delivery systems.

The review from Sarkar et al. highlights how the generation of synthetic designs based on various strategies like sequence truncation, mutation, cyclization and introduction of unnatural amino acids can circumvent the disadvantages of AMPs for their commercial development and exploitation as new therapeutics in the future. In addition, the research article by Brimble et al. shows a concise synthetic method for malacidin A, a lipopeptide specifically active against Gram-positive bacteria, by employing 9-fluorenylmethoxycarbonyl solid-phase peptide synthesis, including late-stage incorporation of the lipid tail, followed by solution-phase cyclisation. The authors also demonstrate the versatility of this synthetic strategy by producing a diastereomeric variant of the lipopeptide and a small library of simplified analogues with variation of the lipid moiety. Recent achievements concerning discovery, distribution, structural and biological characterization of sactipeptides (sulfur-to-alpha carbon thioether cross-linked peptides) as novel compounds endowed with antibacterial, spermicidal and hemolytic properties are then summarized in the work by Chen et al. Moreover, Welch et al. identified several hitherto unknown and unclassified peptides containing motifs of striking similarity to the proline-rich AMP-based DnaK inhibitors and propose a series of new sequences to be included in the PrAMP family of permeable peptides with intracellular targets of DnaK or 70S ribosome. Intriguingly, AMPs as well as small molecules are attractive alternatives for treatment of infectious diseases such as keratitis. This is reviewed by Roy et al. who also discuss about different delivery systems for optimal administration of drugs for treatment of ocular surface infections.

In comparison, the research article from Clements et al. describes the isolation and structural/functional characterization of SM produced by pigmented and non-pigmented *Serratia marcescens* strains. Among these compounds, serratiamolide homologues containing a peptide moiety of two L-serine residues (cyclic or open-ring) linked to two fatty acid chains were found to display activity against *Enterococcus faecalis*. In parallel, Booysen et al. report on SM produced by a bacterium indigenous to South Africa when cultured in aerated broth, non-aerated broth, as well as on the surface of solid media.

Finally, the minireview of Caimano et al. investigated the possibility to increase the dissolution and stability of several inhibitors of the Hedgehog (HH) pathway, whose pharmacological blockade is a promising approach for the clinical management of Medulloblastoma (MB), a highly aggressive paediatric tumour of the cerebellum. The poor pharmacological properties and the low capability to cross the blood–brain barrier of HH inhibitors represents one of the main challenges for treatment of MB. The authors have reported several formulation strategies to improve the bioavailability of the HH inhibitor GlaB, an isoflavone naturally found in the seeds of *Derris glabrescens*, and that is currently patent protected and under preclinical phase for treatment of different HH-dependent cancers.

We believe that the multidisciplinary Research Topic “Secondary Metabolites and Peptides as Unique Natural Reservoirs of New Therapeutic Leads for Treatment of Cancer and Microbial Infections” offers the possibility to open new avenues to identify suitable new lead compounds to fight the alarming challenges of cancer and/or infectious diseases, either when used alone or in combination with traditional drugs.
